# Coexistence of a Novel *OCRL* Variant and a Pathogenic 16p11.2 Deletion in a Patient with Renal and Neurodevelopmental Manifestations Suggestive of Atypical Dent Disease Type 2—A Case Report

**DOI:** 10.3390/jcm15145613

**Published:** 2026-07-17

**Authors:** Christos Chronis, Charikleia Stefanaki, Nikolaos Stergiou, Eleni Kokkou, Periklis Makrythanasis, Konstantina Kosma, Faidon-Nikolaos Tilemis, Maria Tsouprou, Elona Tola, Olga Filippou, Evanthia Botsa

**Affiliations:** 1 Department of Pediatrics, 1st Pediatric Clinic, Agia Sofia Children’s Hospital, 11527 Athens, Greece; 2 Center for Adolescent Medicine and UNESCO Chair in Adolescent Health Care, First Department of Pediatrics, Aghia Sophia Children’s Hospital, School of Medicine, National and Kapodistrian University of Athens, 11527 Athens, Greece; 3 Department of Nephrology, First Department of Pediatrics, Aghia Sophia Children’s Hospital, University of Athens, 11527 Athens, Greece; 4 Unit of Developmental and Behavioral Pediatrics, 1st Department of Pediatrics, National and Kapodistrian University of Athens, 11527 Athens, Greece; 5 Laboratory of Medical Genetics, St. Sophia’s Children’s Hospital, Medical School, National and Kapodistrian University of Athens, 11527 Athens, Greecekkosma50@gmail.com (K.K.);; 6 Department of Genetic Medicine and Development, Medical School, University of Geneva, 1211 Geneva, Switzerland; 7 Biomedical Research Foundation of the Academy of Athens, 11527 Athens, Greece

**Keywords:** *OCRL* gene, Lowe syndrome, dent disease, OCRL gene variants, gene variation, case report

## Abstract

**Background/Objectives**: To date, approximately 360 pathogenic variants of the *OCRL* gene have been reported, including frameshift, substitution, gross inversion, nonsense, and missense mutations. These genetic alterations have been associated with a broad phenotypic spectrum of Lowe syndrome, contributing to considerable variability in disease severity and clinical presentation. Missense variants are typically associated with preserved messenger RNA expression in fibroblasts, whereas more deleterious mutations result in markedly reduced expression of the *OCRL* transcript or protein product. Pathogenic variants in the *OCRL* gene have also been identified in patients with Dent’s disease type 2. **Case presentation**: This case report describes a pediatric male patient with autism spectrum disorder and renal dysfunction, who was found to harbor a variant of uncertain clinical significance in the *OCRL* gene and a pathogenic 16p11.2 chromosomal deletion. The patient, a 12-year-old boy with autism, presented with proteinuria during hospitalization for febrile gastroenteritis and streptococcal infection. Further evaluation revealed focal glomerulosclerosis, tubular calcium phosphate deposits, albuminuria, and hypercalciuria. Ophthalmologic examination additionally demonstrated bilateral lens opacities in the absence of congenital cataracts, as well as hyperopia. The coexistence of these clinical manifestations together with the identified *OCRL* gene variant raises the possibility of an atypical presentation of Dent disease type 2. Nevertheless, continued clinical and genetic follow-up remains warranted to further clarify the pathogenic significance of the detected variant and to establish a definitive diagnosis. **Conclusions**: The uniqueness of this case resides in the coexistence of two independent genetic findings presenting a phenotype-attribution challenge. Furthermore, the identified phenotype may warrant consideration as a possible previously unreported phenotypic presentation situated along the clinical spectrum between Lowe syndrome and Dent disease type 2. The main contribution of this report is to illustrate the interpretative challenges of a concurrent pathogenic 16p11.2 deletion and an *OCRL* variant of uncertain significance in a patient with renal disease and neurodevelopmental features.

## 1. Introduction

The *OCRL* gene is located on the *Xq25–q26.1* region of the X chromosome and consists of 24 exons. It encodes an inositol polyphosphate 5-phosphatase enzyme that localizes to multiple compartments of the endocytic pathway, including the Golgi apparatus, clathrin-coated pits, and early endosomes [1]. The encoded protein is involved in several essential cellular processes, including intracellular signaling, lysosomal trafficking, phagocytosis, cell adhesion and migration, maintenance of cell polarity, ciliogenesis, cytokinesis, and regulation of mechanistic Target of Rapamycin (mTOR) activity, with its functional role varying according to subcellular localization [2]. Approximately 360 *OCRL* gene variations have been identified, including frameshift, substitutions, gross inversions, nonsense, and missense mutations, resulting in a spectrum of Lowe syndrome phenotypes with differing severity. Missense variants typically show normal mRNA expression in fibroblasts, while more severe mutations lead to significantly reduced expression of the *OCRL* transcript or protein. Mutations in the *OCRL* gene are also found in patients with Dent’s disease type 2 [3,4].

This case report describes a 12-year-old male with developmental delay and Autism Spectrum Disorder (ASD), who presented with proteinuria, focal glomerulosclerosis, tubular calcium phosphate deposits, albuminuria, and hypercalciuria during hospitalization for febrile gastroenteritis and streptococcal infection. The patient additionally exhibited bilateral lens opacities in the absence of congenital cataracts, as well as hyperopia. The constellation of these clinical findings, together with the identified *OCRL* variant, suggests an atypical presentation of Dent disease, although continued longitudinal follow-up is required to confirm the diagnosis. This case is particularly notable for the coexistence of a *16p11.2* chromosomal deletion associated with neurodevelopmental disorders and an *OCRL* gene variant suggestive of Lowe syndrome, resulting in overlapping phenotypic features and an atypical presentation consistent with Dent disease type 2. Furthermore, this report underscores the diagnostic challenges associated with phenotypic attribution in the presence of multiple concurrent genetic abnormalities.

## 2. Case Presentation

A 12-year-old male was admitted to the 1st Pediatric Clinic of Aghia Sophia Children’s Hospital in Athens, Greece, due to febrile gastroenteritis and pharyngeal streptococcal infection, subsequently complicated by acute kidney injury. He was naturally conceived and born full-term *via* spontaneous vaginal delivery to non-consanguineous parents, with no perinatal complications. At birth, his weight and height z-scores were within the normal range. His medical history included autism spectrum disorder, diagnosed at the age of three, and he received consistent occupational and speech therapy. Although the patient had a diagnosis of autism spectrum disorder and attended a mainstream school, formal cognitive assessment data were unavailable. Family history was unremarkable. Prior to admission, his routine urinalysis had repeatedly demonstrated low-grade proteinuria. Family medical history was unremarkable.

On admission, during the physical examination, the patient presented with signs of 5% dehydration. Anthropometric assessment revealed increased BMI and short stature. On neurological examination, the patient was alert and fully oriented to space and time. Gross neurological evaluation was, otherwise, unremarkable, apart from generalized hypotonia. Examination of the remaining systems did not reveal any pathological findings. Phenotypic evaluation revealed dysmorphic features, including hypertelorism, a flattened nasal bridge, low-set ears, and short fingers (brachydactyly).

## 3. Diagnostic Assessment

On admission, laboratory evaluation revealed compromised kidney function (serum creatinine = 1.26 mg/dL; blood urea nitrogen (BUN) = 51 mg/dL; Glomerular Filtration Rate (GFR) = 50 m/min/1.73 m^2^; fractional excretion of sodium (FeNa) = 1) and albuminuria, with normal diuresis and muscle enzyme concentrations. Initial management included intravenous hydration, as findings were attributed to prerenal azotemia, related to infection and dehydration. Although clinical improvement was observed in serum creatinine (1.33 mg/dL), both BUN (41 mg/dL) and FeNa > 2 remained elevated, raising suspicions of underlying kidney pathology. Kidney ultrasound showed only slight parenchymal echogenicity (Figure 1a,b). Due to persistent abnormal kidney function and in the context of suspected immune-mediated glomerular involvement at that time, corticosteroids were administered for one month, resulting in normalization of serum creatinine and partial improvement in the biochemical parameters, although albuminuria (1 g/day) persisted, prompting further diagnostic evaluation. After one month of hospitalization, 24 h urine albumin reached 2.5 g, with serum creatinine of 0.75 mg/dL and BUN of 45 mg/dL. Hypercalciuria (4.7 mg/24 h/kg) and increased urine β2-microglobulin levels (max: 64 mg/L) were also documented. The patient was readmitted for a kidney biopsy in collaboration with the Department of Nephrology. Histopathologic findings revealed proximal tubular damage with intratubular calcium phosphate deposition in the medullary parenchyma. Glomerular abnormalities were characterized by focal global glomerulosclerosis and secondary focal segmental glomerulosclerosis, accompanied by vascular changes, including arterial lesions that were considered disproportionate and incompatible with the age of the patient (Figure 2, Figure 3 and Figure 4). Additional investigations identified bilateral lens opacities in the absence of congenital cataract, non-hemodynamically significant aortic insufficiency, and mild pulmonary insufficiency. Otorhinolaryngology (ENT) and neurological examination were unremarkable. MRI of the brain showed symmetrical white matter hyperintensities in the parietal lobes, supraventricular and periventricular white matter, cerebral ventricles, and midbrain, without contrast enhancement (Figure 5a,b).

Considering the patient’s clinical presentation, together with the available laboratory and imaging data and renal biopsy findings, whole-exome sequencing (WES) was undertaken to investigate a possible underlying genetic etiology. Library preparation was performed using the xGen Exome Research v2 kit (Integrated DNA Technologies, IDT), and sequencing was carried out on the NextSeq 500 platform (Illumina). Sequencing generated 99,718,822 total reads, of which 84,393,466 (84.63%) mapped uniquely to the reference genome (hg19/GRCh37), yielding a total of 10.63 Gb of sequence data. The average sequencing coverage was 151×, with a mean target coverage of 143x. Bioinformatic analysis was performed through the VarSome Clinical platform using Burrows–Wheeler Aligner (BWA) for read alignment and the Genome Analysis Toolkit (GATK) for variant calling. A total of 25,403 variants were identified in coding regions.

Variant filtering followed a phenotype-driven strategy. Candidate variants were prioritized based on genes associated with the patient’s phenotype using Human Phenotype Ontology (HPO) and Online Mendelian Inheritance in Man (OMIM) databases. Variants with a minor allele frequency (MAF) > 1% in the Genome Aggregation Database (gnomAD) were excluded. The remaining variants were further filtered according to genomic localization and variant category, with synonymous variants, deep intronic variants, and variants located in the intergenic 3′ untranslated region (UTR), with 5′ UTRs being excluded. Prioritization was subsequently guided by in silico pathogenicity predictions. Variant interpretation and classification were performed according to the recommendations of the American College of Medical Genetics and Genomics (ACMG) and ClinGen guidelines, respectively [5,6].

Genetic testing identified a novel missense variant of uncertain clinical significance in exon 18 of the *OCRL* gene, c.2060T>G (p.Leu687Arg), located within a functional domain of the encoded protein (Table 1). The variant was supported by high-quality sequencing metrics, including a coverage depth of 96x at the variant position, an allelic balance of 1.0, and 98x coverage across the *OCRL* locus. Multiple in silico prediction tools suggested a deleterious effect (REVEL score 0.68, AlphaMissense score 0.993, SIFT score 0, and CADD score 26.7).

Copy number variant (CNV) analysis was performed using ExomeDepth (v1.1.15), an R-based algorithm that detects CNVs from WES data using read-depth comparisons between the test sample and a correlated reference set of unrelated samples processed in the same sequencing batch and matched for sex. CNV calling was based on depth-of-coverage analysis of BAM files using default ExomeDepth parameters. To improve specificity and reduce false-positive calls, only CNVs with a Bayes Factor (BF) ≥ 10 were retained. In addition, thresholds based on the ratio of observed to expected read counts were applied (>0.8 for deletions and <1.1 for duplications), as previously described [7].

This analysis revealed a known pathogenic heterozygous deletion of approximately 524.8 kb in the *16p11.2* chromosomal region [seq[GRCh37] *16p11.2* (29675050_30199897) × 1], encompassing 30 genes, including *PRRT2* and *TBX6* (Table 1). Parental testing and segregation analysis were offered to determine the inheritance pattern of both the *OCRL* variant and the 16p11.2 deletion; however, parental samples were unavailable.

## 4. Conclusions

Lowe syndrome and Dent’s disease type 2 are rare X-linked disorders predominantly affecting male patients. Dent’s disease is characterized by proximal tubular dysfunction and is subclassified into two major types. Type 1 is caused by pathogenic variants in the *CLCN5* gene and is typically confined to renal manifestations, whereas type 2 is associated with variants in the *OCRL* gene and may present with extrarenal features, including mild intellectual disability, ocular involvement, or hypotonia. Pathogenic variants in the *OCRL* gene account for approximately 15% of cases of Dent disease type 2 [8]. Distinguishing the specific type and location of variants within the *OCRL* gene is of clinical importance, as different mutational classes are associated with distinct phenotypic outcomes. Truncating variants, including nonsense and frameshift mutations, particularly those occurring in exon 8 and beyond, have been strongly associated with the classical phenotype of Lowe syndrome, characterized by ocular, neurological, and renal involvement [9,10]. According to GeneReviews (University of Washington), frameshift and nonsense variants associated with Lowe syndrome are typically localized to the middle and distal regions of the *OCRL* gene, particularly within exons 8–23. In contrast, missense variants occurring downstream of exon 8—especially within exons 9–15—may be associated with a broader phenotypic spectrum, including Dent’s disease type 2, which is predominantly characterized by renal tubulopathy [10]. Notably, genotype–phenotype correlations in *OCRL*-related disorders demonstrate considerable variability.

Previous studies have shown that approximately 93% of pathogenic variants associated with Lowe syndrome are located within exons 10–18 and 19–23 of the *OCRL* gene, with a particular clustering in exon 15. Although more than 200 distinct *OCRL* variants have been described, no causative variant is identified in approximately 10–20% of patients with a clinical diagnosis of Lowe syndrome [11]. Recently, variants in exons 1–8 have been linked to Dent’s disease, although genotype–phenotype correlations remain imperfect [12].

The *16p11.2* deletion syndrome is a clinically heterogeneous disorder, characterized by developmental delay, intellectual disability, hyperphagia, obesity, macrocephaly and psychiatric problems. Cases with *16p11.2* duplication syndrome often exhibit a partial ‘mirror phenotype’ including underweight and microcephaly. The *16p11.2* region contains low-copy repeats that predispose to non-allelic homologous recombination, resulting in recurrent copy number variations (CNVs) with variable breakpoints. Recurrent deletions and duplications of 16p11.2 represent some of the most common pathogenic CNVs associated with neurodevelopmental disorders [13]. Depending on the specific genomic loci involved, copy number variants (CNVs) may confer susceptibility to a broad spectrum of neurodevelopmental disorders, frequently characterized by incomplete penetrance and marked phenotypic variability. Recurrent deletions and duplications at the *16p11.2* locus have been implicated in autism spectrum disorder, intellectual disability, motor and developmental delay, dysmorphic phenotypes, and epileptic manifestations, whereas *16p11.2* duplications have additionally been associated with an increased risk of schizophrenia [14]. In the present case, the patient exhibited obesity, which is consistent with the phenotypic spectrum associated with *16p11.2* deletion syndrome.

Several genes within the deleted *16p11.2* chromosomal region have previously been associated with clinical features observed in our patient, including *TBX6*, *QPRT*, and *PRRT2*. The *TBX6* gene is involved in embryonic development, and both human and mouse studies have demonstrated that *TBX6* insufficiency can be associated with kidney dysplasia and congenital anomalies of the kidney and urinary tract [15]. Moreover, within the context of *16p11.2* deletions, *TBX6* has been identified as a major contributor to kidney phenotypes [16]. Additionally, among the deleted genes of the *16p11.2* loci, *PRRT2* has been consistently associated with the physiology of synaptic transmission and neuronal migration, and its deficiency can lead to a wide spectrum of neurodevelopmental disorders, such as autism spectrum disorder [17]. Other genes, within the deleted region, such as *MAPK3*, *KIF22*, *ALDOA* and *KCTD13*, have also been related to neurodevelopmental disorders, but their contribution appears to be less central compared with *PRRT2* [17].

Although pathogenic variation in the *QPRT* gene has been previously implicated in solitary kidney, this manifestation was absent in our patient, suggesting that *QPRT* may nevertheless have contributed to the broader renal phenotype observed in the present case [16].

Based on the current literature, the patient’s renal manifestations, tubular nephropathy, and lens opacities are more consistent with *OCRL*-related disease. The observed white matter hyperintensities may also be related to *OCRL* dysfunction, as similar neuroradiological abnormalities have been reported in patients with *OCRL*-related disorders. However, the clinical significance of these MRI findings remains uncertain, and a causal relationship cannot be established in the present case. In contrast, the presence of autism spectrum disorder and obesity is more consistent with the phenotypic spectrum associated with the *16p11.2* deletion [18].

Renal dysfunction, hypercalciuria, tubular injury, lens opacities, hypotonia, and the observed white matter abnormalities are more consistent with the reported spectrum of *OCRL*-related disease. In contrast, autism spectrum disorder, obesity, and dysmorphic features are more consistent with the established phenotype associated with *16p11.2* deletion syndrome. Nevertheless, because the *OCRL* variant remains classified as a variant of uncertain significance, the relative contribution of each genetic finding to the overall phenotype cannot be definitively determined.

Brain MRI demonstrated symmetrical white matter hyperintensities involving the parietal lobes, supraventricular and periventricular white matter, as well as the midbrain, without contrast enhancement. Neuroimaging abnormalities, including white matter signal changes, have been described in individuals with *OCRL*-related disorders, particularly in patients with classic Lowe syndrome. Although the pathogenic mechanisms underlying these findings remain incompletely understood, they may reflect central nervous system involvement associated with *OCRL* dysfunction. The presence of white matter hyperintensities in our patient, despite his relatively mild clinical phenotype, suggests that neuroradiological abnormalities may occur across the *OCRL* disease spectrum. However, the significance of these findings and their relationship to clinical manifestations require further investigation [19].

The overall phenotype is suggestive of an atypical Dent disease type 2 presentation; however, because the *OCRL* variant remains classified as a variant of uncertain significance, a definitive molecular diagnosis cannot currently be established. Formal cognitive assessment was unavailable. However, the patient attended mainstream education and did not exhibit the severe neurodevelopmental impairment typically reported in patients with classic Lowe syndrome. Both disorders share similar renal manifestations, although these are generally milder in Dent disease type 2 than in classic Lowe syndrome. At the same time, our patient had bilateral lens opacities, without, however, manifesting the severe ophthalmological disorder found in Lowe syndrome. In Dent disease type 2 (*OCRL*-related disorder), the primary defect involves proximal tubular dysfunction, leading to low-molecular-weight proteinuria with progressive albuminuria as tubular reabsorptive capacity declines. Ongoing tubular injury, together with hypercalciuria and phosphate-wasting conditions, promotes intratubular calcium phosphate deposition and medullary nephrocalcinosis, further exacerbating tubular damage. With disease progression, loss of functional nephrons and sustained tubular injury result in compensatory hyperfiltration in remaining glomeruli, leading to secondary focal segmental glomerulosclerosis and focal global glomerulosclerosis. These glomerular lesions are interpreted as secondary adaptive changes rather than primary glomerular disease. Associated vascular changes, including arterial lesions disproportionate to age, likely reflect chronic kidney injury and long-standing hemodynamic stress [20]. In other words, patients with Dent 2 may present a mild form of these symptoms in contrast to the Lowe syndrome. The coexistence of a novel *OCRL* variant and a *16p11.2* chromosomal deletion complicates phenotype attribution in the present case. Variants in the *16p11.2* region are well established as etiological factors in neurodevelopmental disorders [18]. To the best of our knowledge, there is no documentation of any clinically relevant or mechanistic interaction between the *OCRL* variants and the *16p11.2* loci [21]. Both genetic regions are characterized by variable expressivity and incomplete penetrance, which can be influenced by genetic background and modifier genes. However, to date, no evidence supports a modifying effect of *OCRL* variants on *16p11.2* deletion phenotypes, nor an influence of *16p11.2* deletions on the clinical spectrum of *OCRL*-related disorders, rendering this case exceptionally rare.

An important limitation of this study is the absence of parental testing and segregation analysis. Although parental genetic testing was offered, the family declined further investigations because of financial constraints, and parental samples were therefore unavailable. Consequently, the inheritance pattern of both the *OCRL*:c.2060T>G variant and the *16p11.2* deletion could not be determined. Establishing whether either variant occurred de novo or was inherited would have provided important evidence for variant interpretation and genotype–phenotype correlation. Segregation data could have contributed to the classification of the *OCRL* variant, which currently remains a variant of uncertain significance, and would have helped clarify the relative contribution of each genetic finding to the patient’s clinical presentation. Furthermore, orthogonal confirmation of the detected variants could not be performed for the same reason. Therefore, the pathogenic role of the *OCRL* variant and the potential contribution of each genetic finding to the observed phenotype should be interpreted with caution.

Whole-exome sequencing (WES) focuses on the protein-coding regions of the genome and is a cost-effective method for identifying disease-causing variants. However, WES does not detect structural or non-coding variants, such as those in intronic or regulatory regions, which may be clinically relevant. In this context, whole-genome sequencing (WGS) could offer additional insights [22].

Conclusively, this is a case of a novel *OCRL* gene variant coexisting with a *16p11.2* deletion, resulting in a distinct and complex clinical phenotype. This case underscores the importance of cautious phenotype attribution when multiple genetic findings are identified and highlights the need for long-term follow-up in patients with overlapping genetic conditions. Ongoing multidisciplinary follow-up is warranted, given the uncertain prognosis and manifestations over time.

## Figures and Tables

**Figure 1:**
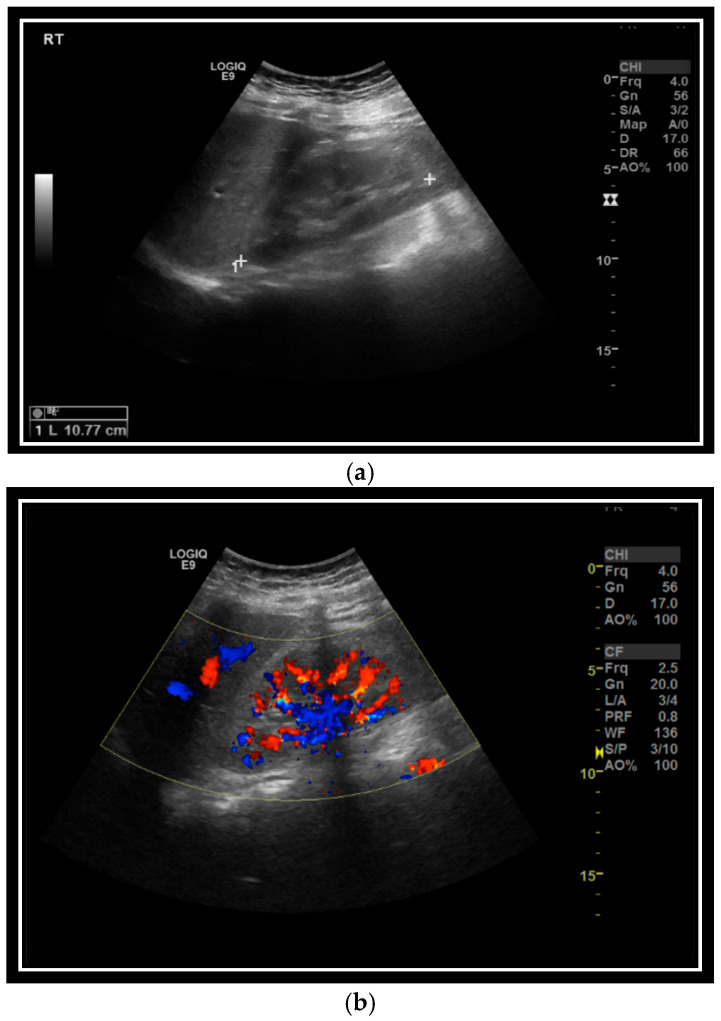
(**a**,**b**) Kidney ultrasound image demonstrating mild diffuse increase in renal parenchymal echogenicity, consistent with minimal parenchymal involvement.

**Figure 2:**
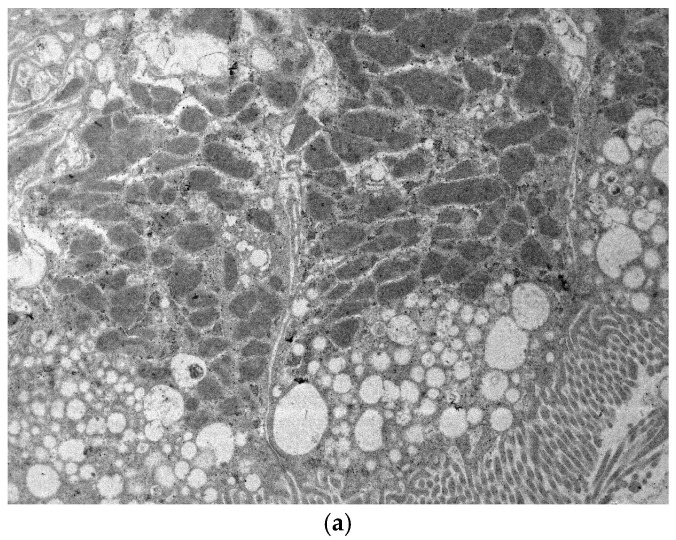

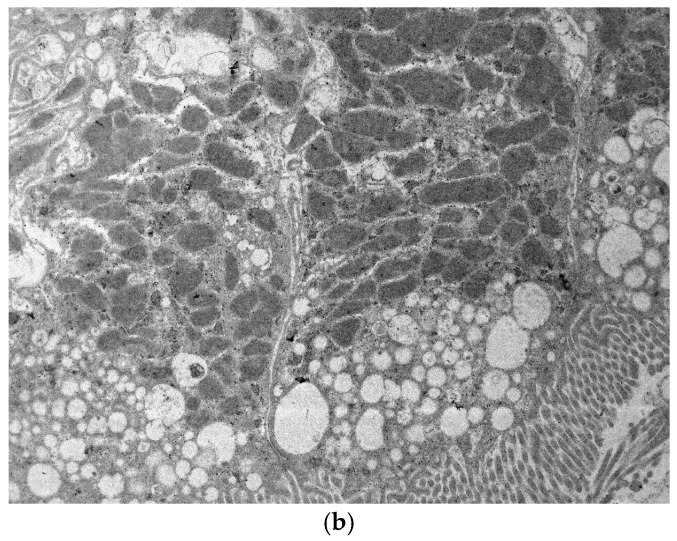
(**a**,**b**) Histopathologic findings revealed proximal tubular damage with intratubular calcium phosphate deposition in the medullary parenchyma. Glomerular abnormalities included secondary focal segmental glomerulosclerosis and arterial lesions incompatible with the patient’s age.

**Figure 3:**
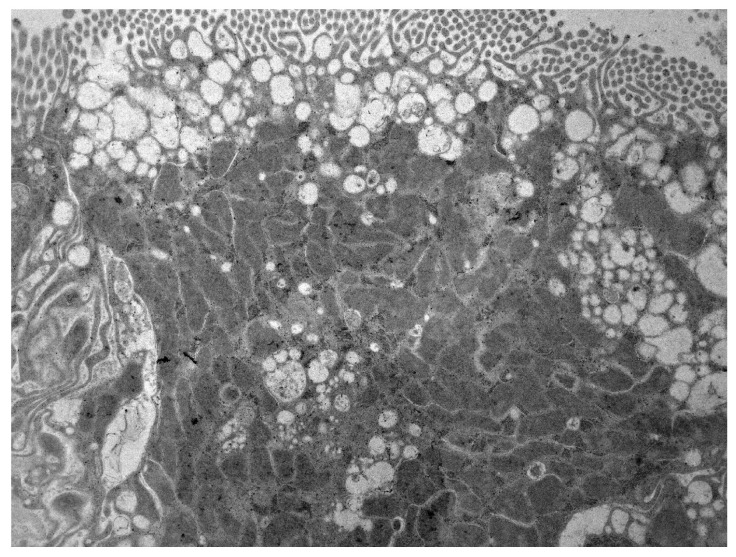
Histopathological finding showing proximal tubules with cytoplasm packed with granules and small vacuoles.

**Figure 4:**
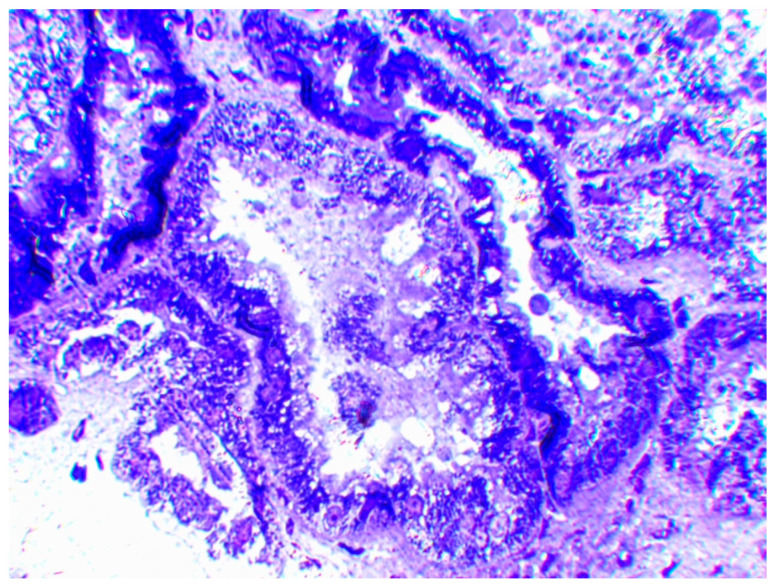
Histopathological evaluation demonstrated the presence of global glomerulosclerosis involving the examined glomerulus.

**Figure 5:**
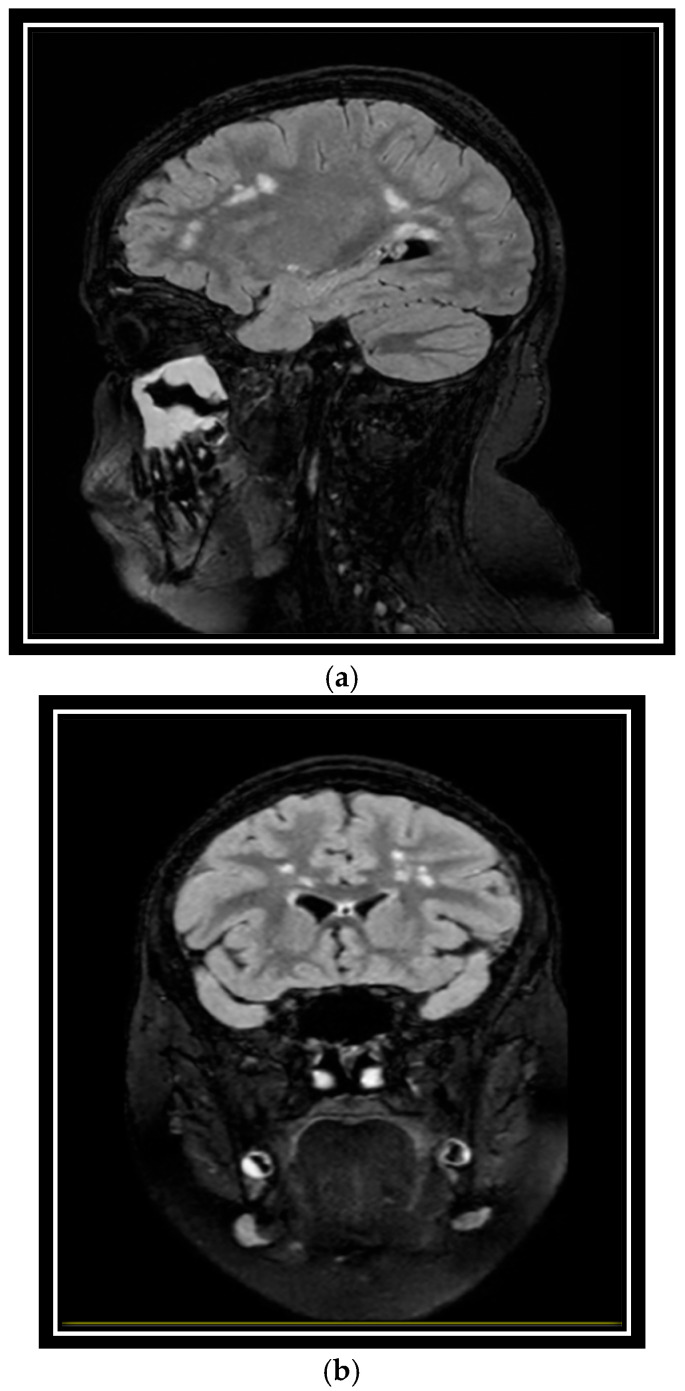
(**a**,**b**) Brain MRI showing incidental, symmetrical white matter hyperintensities involving the parietal lobes, periventricular and subcortical white matter and the midbrain, without contrast enhancement.

**Table 1:** Genetic findings identified in the patient by WES.

Genomic Region/Genes	Variant Coordinates	Allele Status	Inheritance Pattern	Variant Classification (ACMG/ClinGen)
**16p11.2** ***PRRT2***, ***TBX6***, *KIF22*, *ALDOA*, *MAPK3*, *SPN*, *QPRT*, *C16orf54*, *ZG16*, *MAZ*, *PAGR1*, *MVP*, *CDIPT*, *SEZ6L2*, *ASPHD1*, *KCTD13*, *TMEM219*, *TAOK2*, *HIRIP3*, *INO80E*, *DOC2A*, *C16orf92*, *TLCD3B*, *LOC112694756*, *PPP4C*, *YPEL3*, *GDPD3*, *CORO1A)*	(GRCh37/hg19): Copy Number Variant (CNV) seq [GRCh37] 16p11.2 (29675050_30199897) × 1 (Size 524.8 kb)	Heterozygous	Autosomal Dominant	**Pathogenic** (Score 1: 1A, 2A, 2B, 2C, 2H 3B, 4L, 4O)
***OCRL***	(GRCh37/hg19): chrX:128710474T>G **Exon 18** NM_000276.4:**c.2060T>G** **p.(Leu687Arg)**	Hemizygous	X-linked	**Variant of Uncertain Significance (VUS)** (PM2, PP3)

PM2: absent or extremely rare in population databases. PP3: multiple computational tools support a deleterious effect on gene function.

## Data Availability

No new data were created or analyzed in this study. Data sharing is not applicable to this article.
